# Supplementary material for: Relational visual representations underlie human social interaction recognition
Source: Nat Commun. 2023 Nov 11;14:7317. doi: 10.1038/s41467-023-43156-8 (PMC10640586; doi:10.1038/s41467-023-43156-8)
Supplement: Supplementary file 1 — Supplementary Information [file 41467_2023_43156_MOESM1_ESM.pdf]

SUPPLEMENTARY INFORMATION

Relational Visual Representations Underly Human Social Interaction Recognition

Manasi Malik[a], Leyla Isik[a]

[a]Department of Cognitive Science, Johns Hopkins University, Baltimore, MD 21218, United States

SUPPLEMENTARY METHODS

Human Experiment Instructions

We collected human ratings for the PHASE dataset[1] using Prolific. Supplementary Fig. 1 shows a screenshot of the instructions given to the participants.

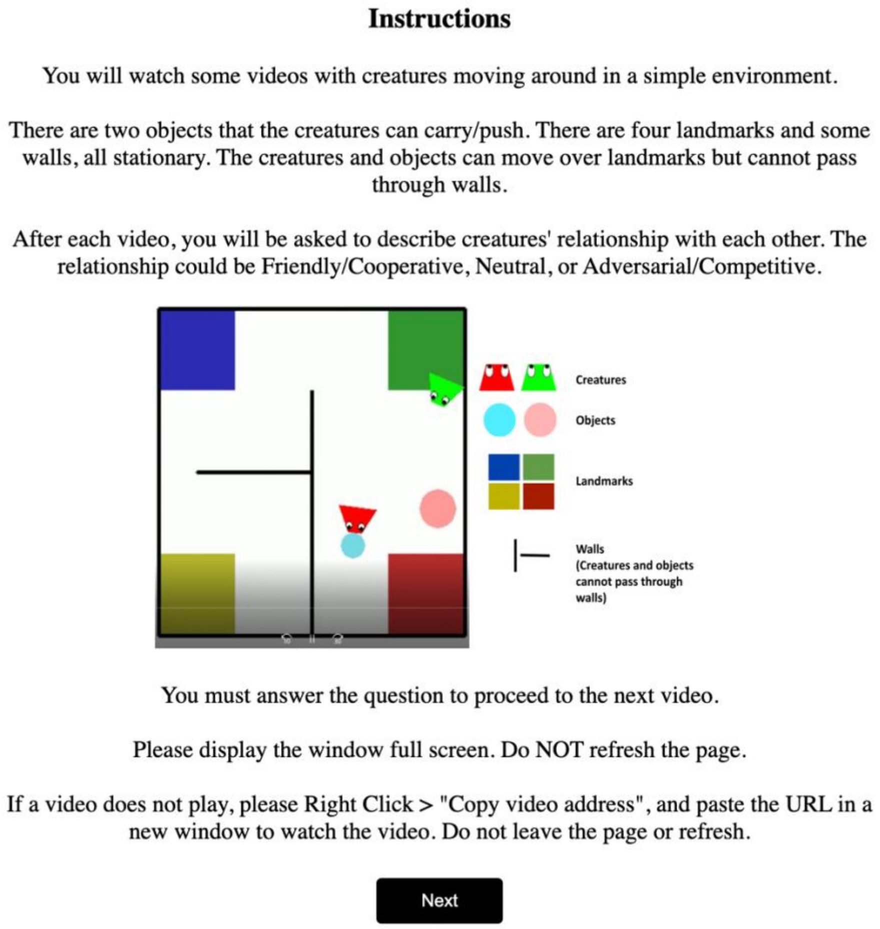

Supplementary Fig. 1: Human Experiment Instructions

Detailed Architectures for SocialGNN and VisualRNN Models

Throughout the supplementary section, we refer to the modified version of SocialGNN that makes predictions based on learned node, rather than edge, representations as "SocialGNN_V", and the original SocialGNN model as "SocialGNN_E".

In this section, we first describe the architecture of SocialGNN_V (Supplementary Fig. 2). Supplementary Fig. 3 then shows the architectures of the SocialGNN models and VisualRNN with detailed steps.

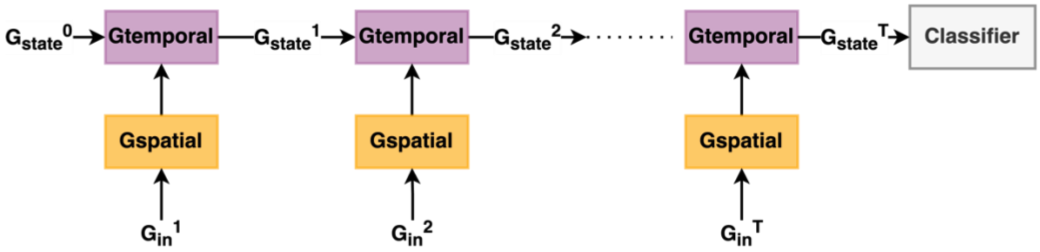

(a) Overall Architecture

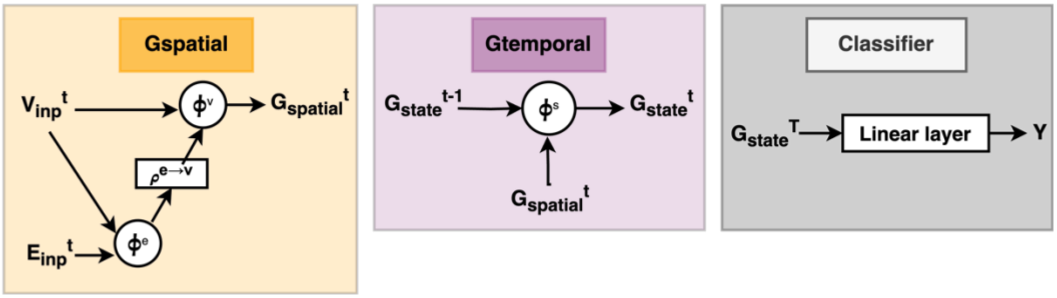

(b) Detailed input/output for SocialGNN_V

Supplementary Fig. 2: SocialGNN_V Architecture. (a) Overall Structure. Here, at each time step, the graph representation of the video frame is passed to the Gspatial module. Gspatial processes the graph and outputs a vector representation, which is passed on to the Gtemporal module. Gtemporal then combines information from previous time steps and the current time step, and the representation at the final time step is passed through a linear classifier to predict the social interaction. (b) Details of Gspatial, Gtemporal, and Classifier modules for SocialGNN_V. The Gspatial module takes in concatenated node representations of nodes on each side of an edge and passes them through a linear layer (ϕ^e). Then, for each node, its features and sum of updated edge representations for all the edges that node is a part of ($\rho^{e\text{->}v}$) are concatenated and passed through an additional linear layer (ϕ^v) that forms an updated node representation. Updated representations for all the nodes are concatenated ($\mathbf{G}_{\mathbf{spatial}}{}^{t}$), appended with context information if present, and then passed to the Gtemporal module where a Long short-term memory (LSTM) unit (ϕ^s) combines information from previous time steps and the current time step to give a new state representation. The representation at the final time step ($\mathbf{G}_{\mathbf{state}}{}^{T}$) is passed through a linear classifier to predict the social interaction (Y).

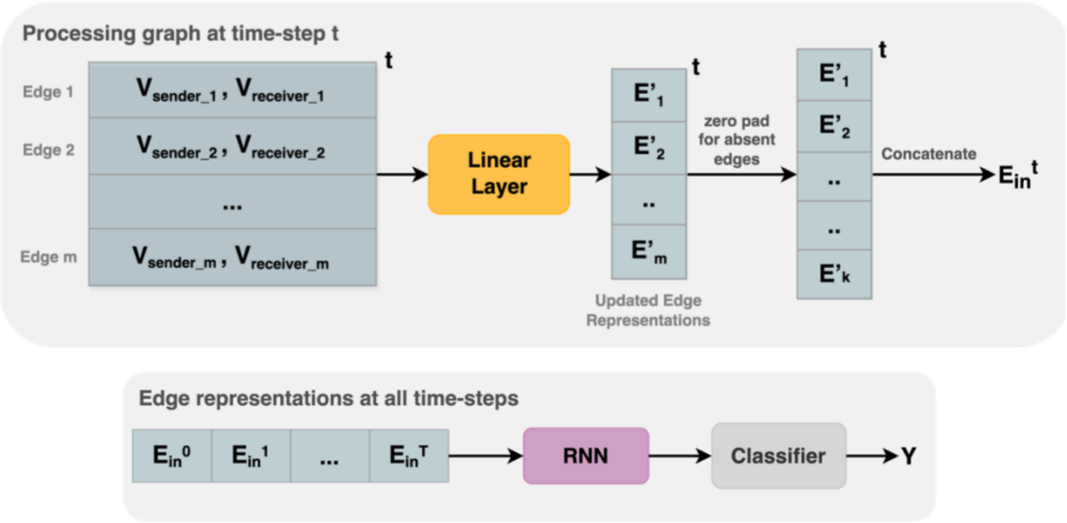

(a) SocialGNN_E Architecture with detailed input/output

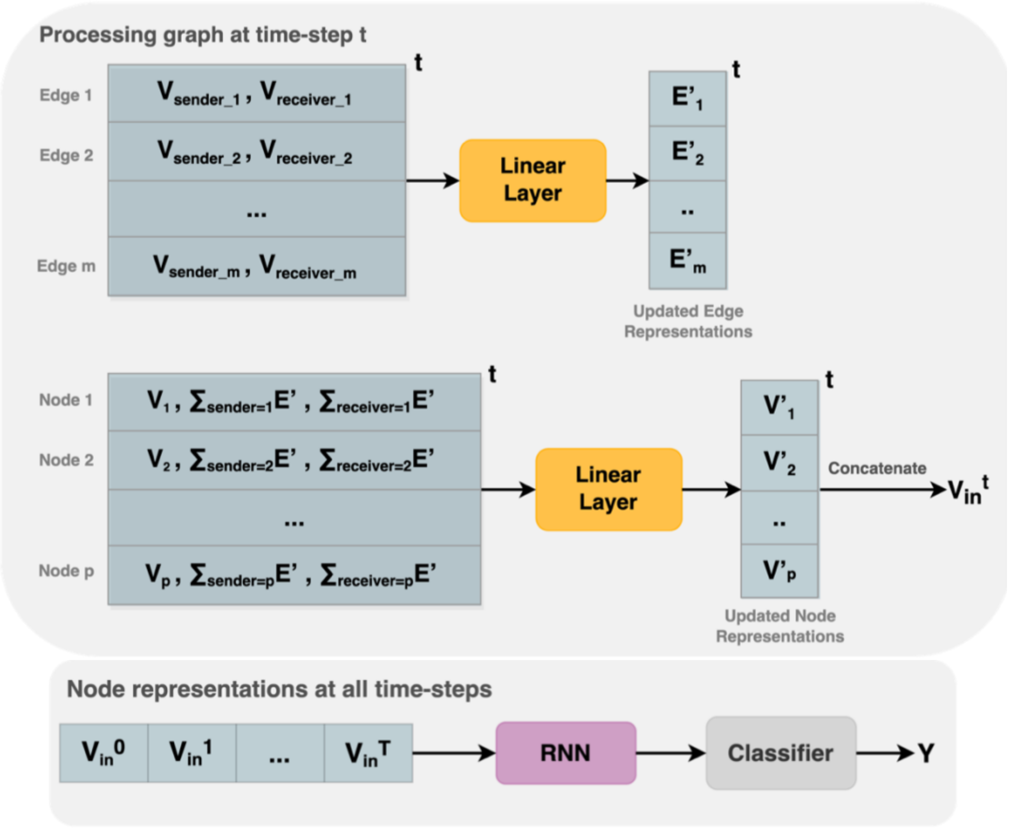

(b) SocialGNN_V Architecture with detailed input/output

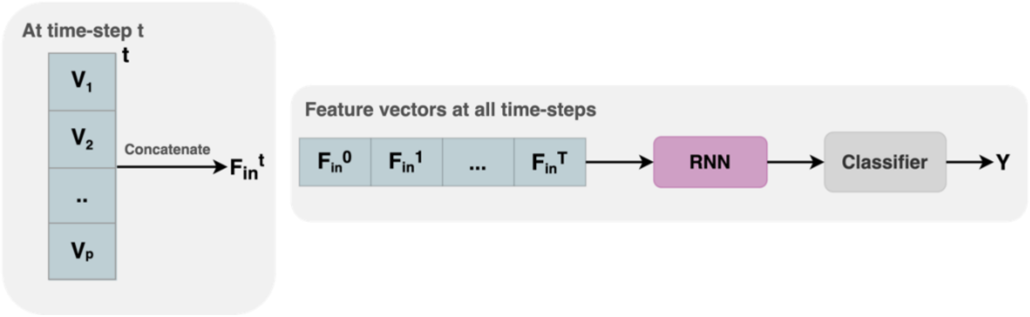

(c) VisualRNN Architecture with detailed input/output

Supplementary Fig. 3: Detailed Architectures for SocialGNN and VisualRNN Models. (a) Edge information is passed to the Gspatial module by concatenating node representations on each side of the edge in ordered sender-receiver pairs ($\mathbf{V}_{sender\_m}^{t}$ and $\mathbf{V}_{receiver\_m}^{t}$ for edge *m)*. These are passed through a linear layer to form the updated edge representations ($\mathbf{E'}_{m}^{t}$), which are then zero-padded, concatenated ($\mathbf{E}_{in}^{t}$), and then passed to the RNN. (b) SocialGNN_V appends each node's features with the updated edge representations for all edges the node belongs to, and then passes that through an additional linear layer. The updated node representations ($\mathbf{V'}_{p}^{t}$) are then concatenated ($\mathbf{V}_{in}^{t}$) and passed through an RNN. (c) VisualRNN takes in the same set of features as SocialGNN, and concatenates them into a feature vector ($\mathbf{F}_{in}^{t}$) that is passed through a similar RNN and classifier as SocialGNN. When context information is present, it is appended to each input edge/node/feature vector at each timestep.

Experimental Settings

We use Python to build and train all our models. For SocialGNN we use the *graph_nets*[2] library. We use the following parameter settings with the architectures described in Fig. 3 and Supplementary Fig. 2. All the models were trained for 150 epochs, with a batch size of 20. Weighted loss used during training has been determined based on the class distribution in the train set: (i) for PHASE, friendly, neutral, adversarial: (1.0, 2.0, 1.0), (ii) Gaze 2-way, social interaction present, absent: (1.0, 1.5), (iii) Gaze 5-way Avert Gaze, Gaze Follow, Joint Att, Mutual Gaze, Single Gaze: (5.69, 4.42, 1.85, 1.66, 1.0). Hyperparameters for all SocialGNN and VisualRNN models were tuned via validation on the training set. The final hyperparameters are listed below.

SocialGNNs:

- ϕ^{e} : Linear layer of size 64 for PHASE, 12 for Gaze
- ϕ^{v} : Linear layer of size 64 for PHASE, 12 for Gaze
- $\rho^{e\rightarrow v}$: Summation function
- ϕ^{s} : LSTM layer of size 16 for PHASE, 6 for Gaze
- Learning Rate: 1e-3
- L2 Regularization Parameter: 0.05/0.01

VisualRNNs:

- $\mathbf{F}_{in}^{t}$ size:
  - VisualRNN (PHASE: 52, Gaze:125)
  - VisualRNN-Rel (PHASE: 64, Gaze:145)
- RNN unit: LSTM layer of size 16 for PHASE, 6 for Gaze
- Learning Rate: 1e-3
- L2 Regularization Parameter: 0.05/0.01

VGG19:

- Learning Rate: 1e-3
- L2 Regularization Parameter: 0.05/0.01

SUPPLEMENTARY NOTE 1

SocialGNN Ablation Study (PHASE)

To understand the success of SocialGNN, we created 2 more modified versions of the SocialGNN models. In SocialGNN_E_onlyagentedges, Gtemporal gets the updated edge representation for only the edge between the two agents whose interaction we are interested in (i.e., no agent-object edges). Similarly, in SocialGNN_V_onlyagents, only node representations of the 2 agents are passed on to Gtemporal at each time step.

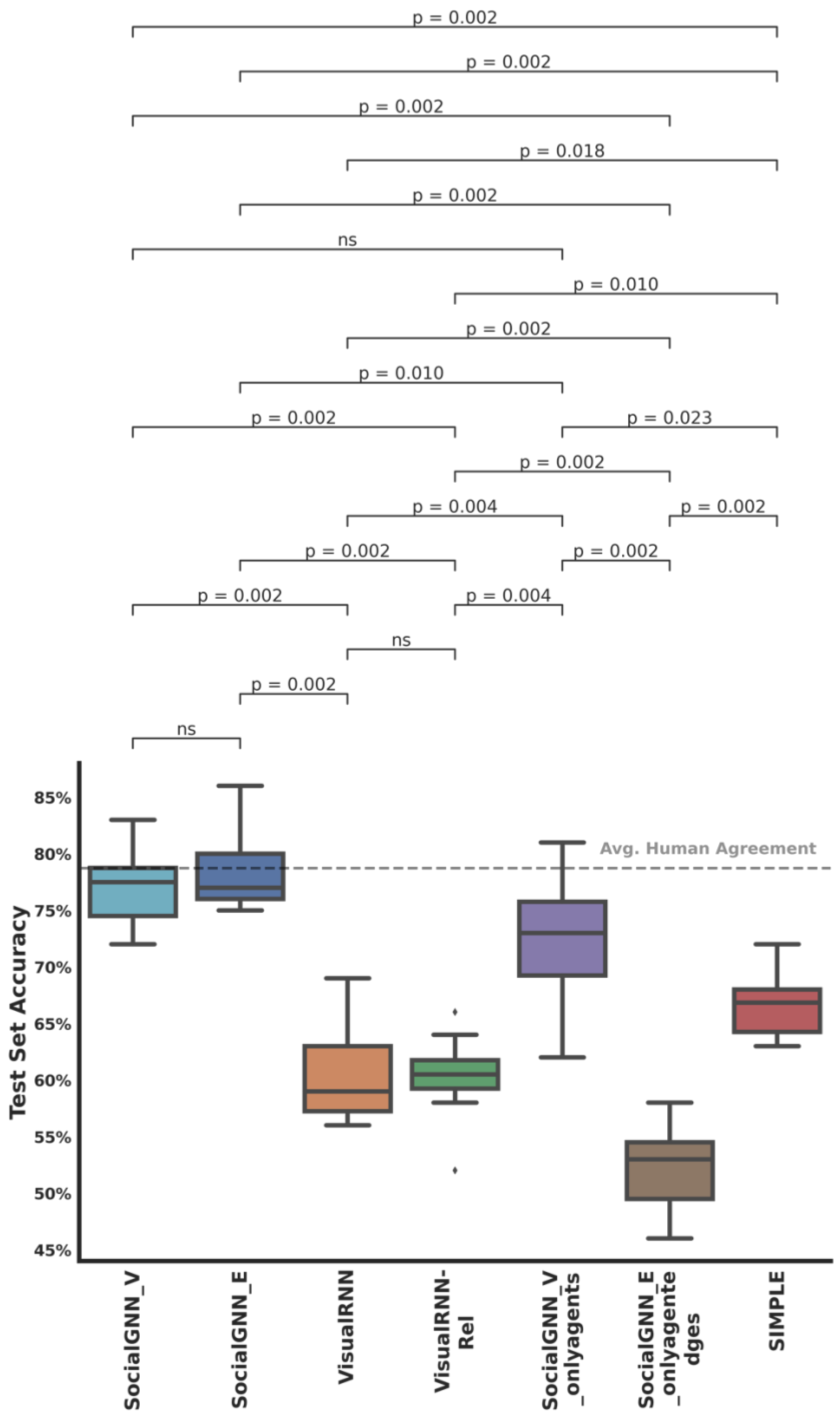

Supplementary Fig. 4: Prediction Accuracy for the type of social interaction (friendly vs. neutral vs. adversarial, chance is 30%) between two agents in the PHASE dataset. For each model, we plot this as a boxplot using accuracies across different train-test splits. The box represents accuracies for 50% of the splits, and the line in the middle of the box is the median. The whiskers represent accuracies outside the middle 50%. Significant differences are denoted with p-values (paired permutation test, n = 10,000 permutations, all two-tailed, Benjamini-Hochberg corrected (since there are a large number of comparisons)).

While SocialGNN_V_onlyagents performs as well as SocialGNN_V, SocialGNN_E_onlyagentedges performs very poorly (Supplementary Fig. 4). This suggests that keeping only the representation of agent-agent edges, loses information about the interactions of the agents with the objects, and this agent-object information is often essential for understanding the social interaction between the two agents. In contrast, while SocialGNN_V_onlyagents does not have object node representations, it still accounts for agent-object edges when updating the agents' node representations. This result further emphasizes the importance of the representation of relations between all the social and non-social entities in a scene to extract social information between agents.

SUPPLEMENTARY NOTE 2

Prediction Performance on Gaze dataset[3]: Confusion Matrices

Supplementary Figs. 5 and 6 show the confusion matrices (averaged across bootstraps) for SocialGNN_V, VisualRNN, VisualRNN-Rel, and VGG19 for 2-way and 5-way classification on the Gaze dataset respectively.

SocialGNN		**Predicted**	
		**Social**	**Non-Social**
**Actual**	**Social**	104.75	47.5
	**Non-Social**	33.1	61.5

VisualRNN		**Predicted**	
		**Social**	**Non-Social**
**Actual**	**Social**	91.2	56.05
	**Non-Social**	48.1	46.5

VisualRNN-Rel		**Predicted**	
		**Social**	**Non-Social**
**Actual**	**Social**	87.15	60.1
	**Non-Social**	46.2	48.4

VGG19		**Predicted**	
		**Social**	**Non-Social**
**Actual**	**Social**	66.65	80.6
	**Non-Social**	41.45	53.15

Supplementary Fig. 5: Averaged Confusion Matrices for Gaze dataset: Predicting the presence of social interaction (2-way classification)

SocialGNN		Predicted				
		Avert Gaze	Gaze Follow	Joint Attention	Mutual Gaze	Single Gaze
Actual	Avert Gaze	7.65	0.65	0.5	5.9	1.65
	Gaze Follow	1.4	14	2.7	1.65	3.15
	Joint Attention	4.65	18.25	7.35	9.1	12.05
	Mutual Gaze	12.7	2.05	2.25	34	5.6
	Single Gaze	14.85	16.6	10.85	22.35	29.95

VisualRNN		Predicted				
		Avert Gaze	Gaze Follow	Joint Attention	Mutual Gaze	Single Gaze
Actual	Avert Gaze	10.05	1.45	1	2.8	1.05
	Gaze Follow	2.4	7.5	7.7	1.55	3.75
	Joint Attention	5.85	14.86	16.25	5.5	9.2
	Mutual Gaze	16.95	9.3	11.55	10.25	8.55
	Single Gaze	15.1	15.7	31.2	15.35	17.25

VisualRNN-Rel		Predicted				
		Avert Gaze	Gaze Follow	Joint Attention	Mutual Gaze	Single Gaze
Actual	Avert Gaze	9.25	1.5	1.5	2.7	1.4
	Gaze Follow	1.9	8.45	7.2	2.55	2.8
	Joint Attention	4.55	17.6	16.05	6.15	7.05
	Mutual Gaze	16.85	11.5	11.2	9.3	7.75
	Single Gaze	13.65	23.5	27	15	15.45

VGG19		Predicted				
		Avert Gaze	Gaze Follow	Joint Attention	Mutual Gaze	Single Gaze
Actual	Avert Gaze	7.4	1.55	2.4	2.7	2.3
	Gaze Follow	3.05	3.85	5.15	3.6	7.25
	Joint Attention	5.9	9.2	12	10.15	14.15
	Mutual Gaze	10.25	8.05	9.3	15.05	13.95
	Single Gaze	13.15	15.75	25.2	17.1	23.4

Supplementary Fig. 6: Averaged Confusion Matrices for Gaze dataset: Predicting the type of gaze communication (5-way classification)

Prediction Performance on PHASE dataset: Confusion Matrices

Supplementary Figs. 7 and 8 show the confusion matrices for SocialGNN_V, VisualRNN, VisualRNN-Rel, and SIMPLE for the held-out test sets in the standard set and the generalization set respectively.

SocialGNN		Predicted		
		**Friendly**	**Neutral**	**Adversarial**
**Actual**	**Friendly**	30.6	2.3	7.2
	**Neutral**	2.2	16.4	1.8
	**Adversarial**	5.5	2.7	31.3

VisualRNN		Predicted		
		**Friendly**	**Neutral**	**Adversarial**
**Actual**	**Friendly**	27.7	5.2	7.2
	**Neutral**	6.5	8	5.9
	**Adversarial**	9.1	5.5	24.9

VisualRNN-Rel		Predicted		
		**Friendly**	**Neutral**	**Adversarial**
**Actual**	**Friendly**	27.7	6.5	5.9
	**Neutral**	5.5	8.6	6.3
	**Adversarial**	7.3	8.2	24

Inverse Planning (SIMPLE)		Predicted		
		**Friendly**	**Neutral**	**Adversarial**
**Actual**	**Friendly**	24.1	4.1	11.9
	**Neutral**	2.9	10	7.3
	**Adversarial**	3.9	3.1	32.5

Supplementary Fig. 7: Confusion Matrices (averaged across bootstraps) for the PHASE Standard Set

SocialGNN		Predicted		
		Friendly	Neutral	Adversarial
Actual	Friendly	32	1	4
	Neutral	5	21	1
	Adversarial	11	2	23

VisualRNN		Predicted		
		Friendly	Neutral	Adversarial
Actual	Friendly	27	4	6
	Neutral	10	4	13
	Adversarial	19	0	17

VisualRNN-Rel		Predicted		
		Friendly	Neutral	Adversarial
Actual	Friendly	30	6	1
	Neutral	18	5	4
	Adversarial	25	4	7

Inverse Planning (SIMPLE)		Predicted		
		Friendly	Neutral	Adversarial
Actual	Friendly	31	1	5
	Neutral	2	21	4
	Adversarial	1	4	31

Supplementary Fig. 8: Confusion Matrices for PHASE Generalization Set

References

1. Netanyahu, A., Shu, T., Katz, B., Barbu, A. & Tenenbaum, J. B. PHASE: PHysically-grounded Abstract Social Events for Machine Social Perception. *Proceedings of the AAAI Conference on Artificial Intelligence* **35**, 845–853 (2021).

2. Battaglia, P. W. *et al.* Relational inductive biases, deep learning, and graph networks. *arXiv preprint arXiv:1806.01261* (2018).

3. Fan, L., Wang, W., Huang, S., Tang, X. & Zhu, S.-C. Understanding human gaze communication by spatio-temporal graph reasoning. in *Proceedings of the IEEE/CVF International Conference on Computer Vision* 5724–5733 (2019).
