# Supplementary material for: Relational visual representations underlie human social interaction recognition
Source: Nat Commun. 2023 Nov 11;14:7317. doi: 10.1038/s41467-023-43156-8 (PMC10640586; doi:10.1038/s41467-023-43156-8)
Supplement: Supplementary file 2 — Reporting Summary [file 41467_2023_43156_MOESM2_ESM.pdf]

nature portfolio

Corresponding author(s): Manasi Malik, Leyla Isik

Last updated by author(s): Oct 27, 2023



# Reporting Summary

Nature Portfolio wishes to improve the reproducibility of the work that we publish. This form provides structure for consistency and transparency in reporting. For further information on Nature Portfolio policies, see our Editorial Policies and the Editorial Policy Checklist.

## Statistics

For all statistical analyses, confirm that the following items are present in the figure legend, table legend, main text, or Methods section.

| n/a | Confirmed | |
|---|---|---|
| ☐ | ☒ | The exact sample size ($n$) for each experimental group/condition, given as a discrete number and unit of measurement |
| ☐ | ☒ | A statement on whether measurements were taken from distinct samples or whether the same sample was measured repeatedly |
| ☐ | ☒ | The statistical test(s) used AND whether they are one- or two-sided<br>*Only common tests should be described solely by name; describe more complex techniques in the Methods section.* |
| ☒ | ☐ | A description of all covariates tested |
| ☐ | ☒ | A description of any assumptions or corrections, such as tests of normality and adjustment for multiple comparisons |
| ☐ | ☒ | A full description of the statistical parameters including central tendency (e.g. means) or other basic estimates (e.g. regression coefficient) AND variation (e.g. standard deviation) or associated estimates of uncertainty (e.g. confidence intervals) |
| ☐ | ☒ | For null hypothesis testing, the test statistic (e.g. $F$, $t$, $r$) with confidence intervals, effect sizes, degrees of freedom and $P$ value noted<br>*Give P values as exact values whenever suitable.* |
| ☒ | ☐ | For Bayesian analysis, information on the choice of priors and Markov chain Monte Carlo settings |
| ☒ | ☐ | For hierarchical and complex designs, identification of the appropriate level for tests and full reporting of outcomes |
| ☐ | ☒ | Estimates of effect sizes (e.g. Cohen's $d$, Pearson's $r$), indicating how they were calculated |

*Our web collection on statistics for biologists contains articles on many of the points above.*

## Software and code

Policy information about availability of computer code

| | |
|---|---|
| Data collection | Prolific online platform was used get behavior data supporting our findings. |
| Data analysis | We used custom code in Python3. The analysis code and instructions to use the code are available on Github: https://github.com/Isik-lab/SocialGNN v1.0.2 |

For manuscripts utilizing custom algorithms or software that are central to the research but not yet described in published literature, software must be made available to editors and reviewers. We strongly encourage code deposition in a community repository (e.g. GitHub). See the Nature Portfolio guidelines for submitting code & software for further information.

## Data

Policy information about availability of data

All manuscripts must include a data availability statement. This statement should provide the following information, where applicable:

- Accession codes, unique identifiers, or web links for publicly available datasets
- A description of any restrictions on data availability
- For clinical datasets or third party data, please ensure that the statement adheres to our policy

Human behavior data included in this paper, along with the processed annotations from the PHASE and Gaze datasets are available on Github: (https://github.com/Isik-lab/SocialGNN/tree/main). The original videos and annotations can be downloaded from https://tshu.io/PHASE/ for the PHASE dataset, and requested from https://github.com/LifengFan/Human-Gaze-Communication for the Gaze dataset. Source data for all figures are provided with the paper.

# Research involving human participants, their data, or biological material

Policy information about studies with human participants or human data. See also policy information about sex, gender (identity/presentation), and sexual orientation and race, ethnicity and racism.

| | |
|---|---|
| Reporting on sex and gender | Sex and gender were not considered in the study design i.e. we did not differentiate participants based on sex or gender in our analyses. Sex and gender were self-reported in a questionnaire before the study. |
| Reporting on race, ethnicity, or other socially relevant groupings | No socially constructed or socially relevant categorization variables were used in our manuscript. |
| Population characteristics | See above |
| Recruitment | We recruited participants via an online platform open to everyone across the world.<br><br>Since the study was done with instructions and response options in English, all participants would need to know how to read English and that could be a potential bias. However, our study had a diverse set of participants from many countries so we expect minimal impact on our findings. |
| Ethics oversight | All studies detailed here received ethical approval from the Johns Hopkins Homewood Institutional Review Board and complied with all relevant ethical regulations. Informed consent was obtained from all participants before the experiment. |

Note that full information on the approval of the study protocol must also be provided in the manuscript.

# Field-specific reporting

Please select the one below that is the best fit for your research. If you are not sure, read the appropriate sections before making your selection.

☐ Life sciences ☒ Behavioural & social sciences ☐ Ecological, evolutionary & environmental sciences

For a reference copy of the document with all sections, see nature.com/documents/nr-reporting-summary-flat.pdf

# Behavioural & social sciences study design

All studies must disclose on these points even when the disclosure is negative.

| | |
|---|---|
| Study description | We proposed and analyzed different models of human social interaction judgments using both animated and natural videos. For the animated dataset, we collected human responses using Prolific. Participants performed a social interaction judgment task where they provide relationship ratings (qualitative) for observed agents. We then quantify these ratings by calculating inter-subject agreement. For the natural videos, we use human annotations provided with the dataset. For both animated and natural videos we quantitatively evaluate the models' match to human behavior data. |
| Research sample | We used Prolific to get human ratings on the animated videos. We had 318 (mean age = 28, age range = 18-68, Sex: 157 Female, 148 Male, 13 Unspecified) participants for the main dataset and 103 (mean age = 37, age range = 19-72, Sex: 55 Female, 46 Male, 2 Unspecified) for the generalization set. The participants were from from multiple countries. Since we're studying a universal human ability - how adult humans make inferences from visual scenes, we wanted to get a wide sample from humans aged 18+, irrespective of sex, and nationality.<br><br>For the natural videos dataset, we used existing annotations provided with their dataset (Fan et al., 2019). |
| Sampling strategy | The sampling procedure was Random as the study was distributed to all available participants on Prolific. No statistical method was used to predetermine sample size. The sample size was determined such that we had at least 10 ratings per video. The number of ratings per video was chosen based on work from others collecting similar ratings with the same dataset (e.g., Netanyahu et al. 2021) |
| Data collection | Data collection was conducted entirely online through the Prolific platform, which the participants accessed using their personal computing devices. The study was designed to be self-administered by participants, and the data collection was remote, without in-person interactions or observers. Given the remote and independent nature of online participation, we did not have direct control or visibility over the participant's physical environment and cannot confirm whether others were present in the room with the participant while they completed the study. The researcher was blind to the experimental condition and/or study hypothesis during data collection. |
| Timing | Data for the main-set animated videos was collected from 16th Aug 2021 to 15th Sept 2021. For the generalization-set animated videos, data was collected between 20th Mar 2022 and 31st Mar 2022. |
| Data exclusions | We excluded 77 and 36 participants from the main-set experiment and generalization-set experiment respectively since they failed on our catch trials or had incomplete responses. |

| | |
|---|---|
| Non-participation | 11 participants and 6 participants declined participation from the main-set experiment and generalization-set experiment respectively. |
| Randomization | We split each dataset randomly into subsets of 20 videos. Each participant was randomly assigned a subset. The order of presentation of these videos was randomized. |

# Reporting for specific materials, systems and methods

We require information from authors about some types of materials, experimental systems and methods used in many studies. Here, indicate whether each material, system or method listed is relevant to your study. If you are not sure if a list item applies to your research, read the appropriate section before selecting a response.

## Materials & experimental systems

| n/a | Involved in the study |
|---|---|
| ☒ | ☐ Antibodies |
| ☒ | ☐ Eukaryotic cell lines |
| ☒ | ☐ Palaeontology and archaeology |
| ☒ | ☐ Animals and other organisms |
| ☒ | ☐ Clinical data |
| ☒ | ☐ Dual use research of concern |
| ☒ | ☐ Plants |

## Methods

| n/a | Involved in the study |
|---|---|
| ☒ | ☐ ChIP-seq |
| ☒ | ☐ Flow cytometry |
| ☒ | ☐ MRI-based neuroimaging |

## Plants

| | |
|---|---|
| Seed stocks | N/A |
| Novel plant genotypes | N/A |
| Authentication | N/A |
