# Supplementary material for: Relational visual representations underlie human social interaction recognition
Source: Nat Commun. 2023 Nov 11;14:7317. doi: 10.1038/s41467-023-43156-8 (PMC10640586; doi:10.1038/s41467-023-43156-8)
Supplement: Supplementary file 3 — Description of Additional Supplementary Files [file 41467_2023_43156_MOESM3_ESM.pdf]

Title: Supplementary Movie 1:
Description: Example of a video from the PHASE dataset where SocialGNN and the Inverse Planning Model disagree in their predictions (‘friendly’ and ‘adversarial’ respectively), and SocialGNN’s prediction matches the mode human judgement. (‘friendly’).

Title: Supplementary Movie 2:
Description: Example of a video from the PHASE dataset where SocialGNN and the Inverse Planning Model disagree in their predictions ('friendly’ and 'neutral’ respectively), and Inverse Planning model’s prediction matches the mode human judgement. ('neutral’).

Title: Supplementary Movie 3:
Description: Example of a video from the PHASE dataset where SocialGNN and the Inverse Planning Model agree in their predictions, and the predictions match the mode human judgement (‘adversarial’). Supplementary Video 4: Example of a video from the PHASE dataset where SocialGNN and the Inverse Planning Model disagree in their predictions ('adversarial’ and ‘neutral’ respectively), and neither match the mode human judgement ('friendly’).
